# Application of the FISH method and high-density SNP arrays to assess genetic changes in neuroblastoma—research by one institute

**DOI:** 10.3389/abp.2024.12821

**Published:** 2024-07-10

**Authors:** Dorota Winnicka, Paulina Skowera, Magdalena Stelmach, Borys Styka, Monika Lejman

**Affiliations:** Independent Laboratory of Genetic Diagnostics, Medical University of Lublin, Lublin, Poland

**Keywords:** neuroblastoma, childhood tumours, MYCN amplification, high-density SNP arrays, FISH

## Abstract

Neuroblastoma is the most common extracranial solid tumor in children. Amplification of the MYCN gene has been observed in approximately 20%–30% of tumors. It is strongly correlated with advanced-stage disease, rapid tumor progression, resistance to chemotherapy and poor outcomes independent of patient age and stage of advanced disease. MYCN amplification identifies high-risk patients. To assess neuroblastoma tumors with MYCN amplification we used paraffin-embedded tissue sections in 57 patients and intraoperative tumor imprints in 10 patients by fluorescence *in situ* hybridization (FISH). Positive results for *MYCN* amplification have been observed in twelve patients’ paraffin-embedded tissue sections and in three patients’ intraoperative tumor imprints, which represents 22.4% of all patients tested in the analysis. Fluorescence *in situ* hybridization is a highly sensitive and useful technique for detecting MYCN amplification on paraffin-embedded tissue sections of neuroblastoma tumors and intraoperative tumor imprints thus facilitating therapeutic decisions based on the presence or absence of this important biologic marker. The presence of structural changes, regardless of MYCN gene amplification status, influences the clinical behavior of neuroblastoma. High-Density SNP Arrays have emerged as the perfect tools for detecting these changes due to their exceptional accuracy, sensitivity and ability to analyze copy number and allele information. Consequently, they are proven to be highly valuable in the genomic diagnosis of immature neuroectodermal tumors.

## Introduction

Neuroblastoma is a malignant tumor in children aged 0–15 years. It accounts for 8%–10% of all childhood tumors, with 80% affecting children under the age of 5 years and approximately 15% representing all cancer deaths in children (Aygun, 2018; Swift et al., 2018). Approximately 60–70 of the new incidences of neuroblastoma are diagnosed each year in Poland (Whittle et al., 2017).

Neuroblastoma derives from primitive cells of the sympathetic nervous system. It occurs in the retroperitoneum, posterior mediastinum, neck, or pelvis. The clinical symptoms in neuroblastoma depend on the tumor mass, the extent of metastasis and catecholamine and vasoactive intestinal peptide secretion by the tumor cells. The diagnosis of neuroblastoma is based on histopathological results and the presence of tumor cells in the bone marrow accompanied by elevated levels of urinary catecholamines. In addition to clinical, histopathological and laboratory data, genetic test results are crucial in determining prognosis (Adamkiewicz-Drożyńska, 2006; Schulte et al., 2018).

The most common cytogenetic changes include deletions of chromosome 1p, amplification of the oncogene *MYCN*, ploidy changes, gains of chromosome arm 17q and deletion of 11q in tumor cells. Genetic abnormalities are very powerful predictors of response to therapy and outcome and provide important information to guide optimal patient management (Bhat and McGregor, 2017; Lim et al., 2020).

The *MYCN* gene is a cellular protooncogene of the MYC family. *MYCN* maps to the 2p24 chromosome. Structural alterations leading to amplification can manifest in the form of double minute chromosomes (dmins) or homogeneous staining regions (HSRs). Dmins represent extra chromosomal material, while HSRs denote duplicate material within chromosomes. Dmins are commonly observed in tumor-derived cells, whereas HSRs are more prevalent in cell lines. However, without traditional karyotype analysis, we are unable to precisely determine what type of changes we are dealing with (Van Noesel and Versteeg, 2004; Jeison et al., 2010).

MYCN is an oncogenic transcription factor involved in the regulation of proliferation, transformation, differentiation and apoptosis (Swift et al., 2018).

*MYCN* amplification is detected in 20%–30% of neuroblastoma patients (Yue et al., 2017; Otte et al., 2020). It is strongly correlated with advanced disease stage, rapid tumor progression, resistance to chemotherapy and poor outcomes. The *MYCN* amplification test result is included in medical protocols around the world, it is an independent indicator in the assessment of the patient outcome and it is one of the qualifying factors for a group requiring the most aggressive treatment (Brodeur et al., 1985; Huang and Weiss, 2013; Ahmed et al., 2017; Kaczówka et al., 2018; Croteau et al., 2021). *MYCN* amplification is present in 38% of patients with stages 3 and 4 (Ahmed et al., 2017) but only in 5%–10% of patients with stages 1, 2, and 4 s (Morandi, 2022). The level of *MYCN* gene amplification is necessary to avoid either under- or over-treating patients (Lee et al., 2018). The gold standard in the assessment of *MYCN* amplification is fluorescence *in situ* hybridization on paraffin-embedded tissue sections and intraoperative tumor imprints.

The independent factors that predict the outcome of treatment are: tumor histology, level of *MYCN* amplification and chromosomal copy number changes (Chicard et al., 2016; Lerone et al., 2021). In addition to these two main genetic markers, a number of segmental aberrations involving different chromosomes have also been described that, in addition to *MYCN* amplification, have a strong effect on the development of neuroblastoma, making it more aggressive and resistant to treatment (Lerone et al., 2021). Currently, the diagnosis of neuroblastoma also relies on molecular methods, such as microarrays, but it is not always possible to isolate good-quality DNA from paraffin-embedded tissue sections. An alternative method to obtain good-quality DNA is the intraoperative collection of tumor scrap from which DNA isolation is performed directly.

SNP array analysis provides comprehensive detection of the presence of deletions and duplications involving chromosomal fragments and allows for simultaneous assessment of DNA copy number.

SNP array analysis can also detect loss of heterozygosity (LOH). Studies of neuroblastoma cells have identified LOH in several chromosomal regions, of which LOH 1p and 4p correlate with poor patient outcomes.

In addition to these relatively large genomic aberrations, deletion aberrations of single genes, parts of genes or point mutations e.g., *TERT*, *ALK*, *ATRX,* and *ARID1A*, have recently been described as having unfavorable clinical outcomes and implications for clinical management (Brunner et al., 2016).

The aim of this study was to demonstrate the effectiveness of the FISH method for the evaluation of *MYCN* amplification on paraffin-embedded tissue sections and intraoperative tumor imprints together with HD SNP array analysis in neuroblastoma patients.

## Materials and methods

The study materials consisted of 57 paraffin-embedded tissue sections with freshly removed tumors and 10 intraoperative tumor imprints, along with archival sections from 67 patients with an established clinical diagnosis of neuroblastoma (31 little girls and 36 little boys; median age: 3 years 2 months). All of the children were admitted to the Department of Pediatric Hematology, Oncology and Transplantology, Children’s University Hospital, Lublin, Poland between 1994 and 2023.

The collected clinical data included the patient`s age at diagnosis, stage according to the INSS (International Neuroblastoma Staging System) criteria (Park et al., 2017), tumor location, histopathological diagnosis of the examined tissues and *MYCN* amplification [Table 1]. Detailed patient data can be found in the Supplementary Material. All studies were performed between 1994-2023.

**TABLE 1:** Patient characteristics.

	Criterium	Number
Age at diagnosis	<18 m >18 m	31 36
Diagnosis	NB GNB GN ND	52 11 1 3
INSS risk group	1 2 3 4s 4	7 3 24 3 30
Primary site	Abdomen Adrenal glands Mediastinum Pelvis Cervical	22 25 11 7 2
*MYCN* amplification	Yes No	15 52

### FISH method

We used the Pretreatment Kit (Vysis) and the LSI N-MYC (2p24) SpectrumGreen/CEP 2 SpectrumOrange Probe (Vysis) to estimate *MYCN* amplification.

Paraffin-embedded tissue blocks were cut into 4-micron thick sections on silanized slides and they were baked at 56°C overnight. Slides were soaked twice in xylene for 10 min and dehydrated in ethanol at 100%, 96%, 80%, and 70% for 2–5 min in each solution. Slides were then immersed in 0.2 N hydrochloric acid for 20 min and rinsed in water and wash buffer for 3 min in each of them. The slides were immersed in Pretreatment Solution at 80°C for 30 min and they were washed twice in 2xSSC for 5 min.

In the next step, the tissue sections were digested in protease solution at 37°C for 30 min and then the slides were immersed in 2xSSC cooled to 5°C.

The slides were then fixed in 10% formalin for 10 min. Fixed tissue, washed twice in 2xSSC and dried on a hot plate at 45°C–50°C.

Slides with intraoperative tumor imprints were immersed in a fixative prepared by mixing 3 parts methanol and 1 part acetic acid for 30 min. The slides were then dried at room temperature for 24 h.

To detect the presence of *MYCN* gene amplification, on paraffin-embedded tissue sections and intraoperative imprints of neuroblastoma tumors, we used fluorescence *in situ* hybridization (FISH) on interphase nuclei. Molecular cytogenetics uses the FISH method to identify specific DNA sequences on chromosomes using DNA probes that are directly labeled by the fluorophore. The labeled probe and the target DNA are denatured in the first step of this method. Hybridization is the second step of the FISH method. Combining the denatured probe and target DNA allows for the annealing of complementary DNA sequences.

We used 1 µL of direct fluorochrome-labeled, dual color DNA Probe for the *MYCN* gene locus (2p24) and α-satellite DNA identifying the centromeric region of chromosome 2 (Vysis LSI N-MYC (2p24) SpectrumGreen/CEP 2 SpectrumOrange Probe) combined with the 7 µL Hybridization Buffer and 2 µL water. The probe was placed on a slide, then covered with a coverslip and secured with rubber cement. The slides were incubated at 72°C for 5 min to denature the DNA. Hybridization was performed for 14–18 h at 37°C.

**FIGURE 1:**
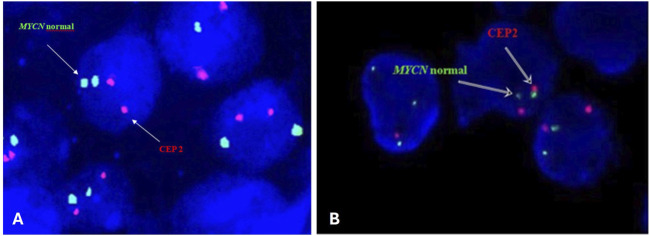
Representative FISH image on paraffin-embedded tissue section **(A)** and intraoperative tumor imprints **(B)** using the Vysis LSI N-MYC (2p24) SpectrumGreen/CEP 2 SpectrumOrange Probe from a neuroblastoma tumor showing normal interphase nuclei captured with Applied Spectral Imaging- ASI-Israel at ×100 magnification on the Nikon epifluorescence microscope.

The slides were subsequently immersed in post-hybridization wash buffer at room temperature for 15–20 min until the coverslip fell off. Then they were immersed in post-hybridization wash buffer warmed to 72°C for 5 min. The cells were counterstained with DAPI. The signals were evaluated by eye or captured with Applied Spectral Imaging- ASI- Israel at ×100 magnification on the Nikon epifluorescence microscope equipped with a 100-W mercury arc lamp and an appropriate set of filters (single filters for FITC and TexasRed fluorochromes and a triple bandpass filter for FITC/TR/DAPI). A minimum of 200 nuclei was evaluated for each of the *MYCN* and CEP2 loci. Nuclei with two visible red and two green signals were scored as negative for *MYCN* amplification (Figure 1). According to the guidelines, the cutoff point for *MYCN* amplification was set at 10 signals (amplification ≥10; gain <10) only in the presence of disomy of chromosome 2. In the presence of polysomy of chromosome 2 in tumor cells, confirmation of *MYCN* gene amplification was based on the guideline that the number of tested signals must exceed that of control signals by at least four times (Ambros et al., 2009; Winnicka et al., 2020).

The study was approved by the Ethics Committee of the Medical University of Lublin (KE-0254/229/2012).

### HD SNP array method

In eight patients, we isolated DNA from tumor tissue and performed microarray testing using the CytoScan HD array to evaluate additional cytogenetic abnormalities. In one case, the quality of the DNA did not allow for the array procedure.

Genomic DNA was isolated with the QIAamp DNA Blood Mini Kit (Qiagen, Hilden, Germany) according to the manufacturer’s protocols. DNA samples were stored at −20°C until the next step. The concentration and quality of DNA were determined using a spectrophotometric method (NanoDrop 8000; Thermo Fisher Scientific, Waltham, MA, United States). In total, 250 ng of genomic DNA was used in accordance with the manufacturer’s protocols. Microarray testing was performed with the use of a CytoScan HD array [2,670,000 markers, including 750,000 SNP and 1,900,000 non-polymorphic copy number variant (CNV) markers] (Applied Biosystems, part of Thermo Fisher Scientific). All subsequent steps and data analysis were performed as described previously by Lejman et al. (2020). The scanned data file was analyzed with Chromosome Analysis Suite v 3.3 (ChAS; Thermo Fisher Scientific) using the CRCh37 (hg19) reference genome.

## Results

### Results of *MYCN* amplification by fluorescence *in situ* hybridization (FISH)

Sixty-seven patients participated in this study. Thirty-four (50.7%) of patients had localized neuroblastoma (1–3 stages), 4.5% stage 4S, and 44.8% stage 4 disease. Forty-seven (70.1%) of patients had an abdominal/adrenal mass at diagnosis. Thirty-one (46.3%) patients were <18 months of age, and thirty-six (53.7%) patients were >18 months of age. Of 67 patient samples, including 52 neuroblastomas, 11 ganglioneuroblastomas, and 1 ganglioneuroma, 3 samples had no definitive diagnosis.

**FIGURE 2:**
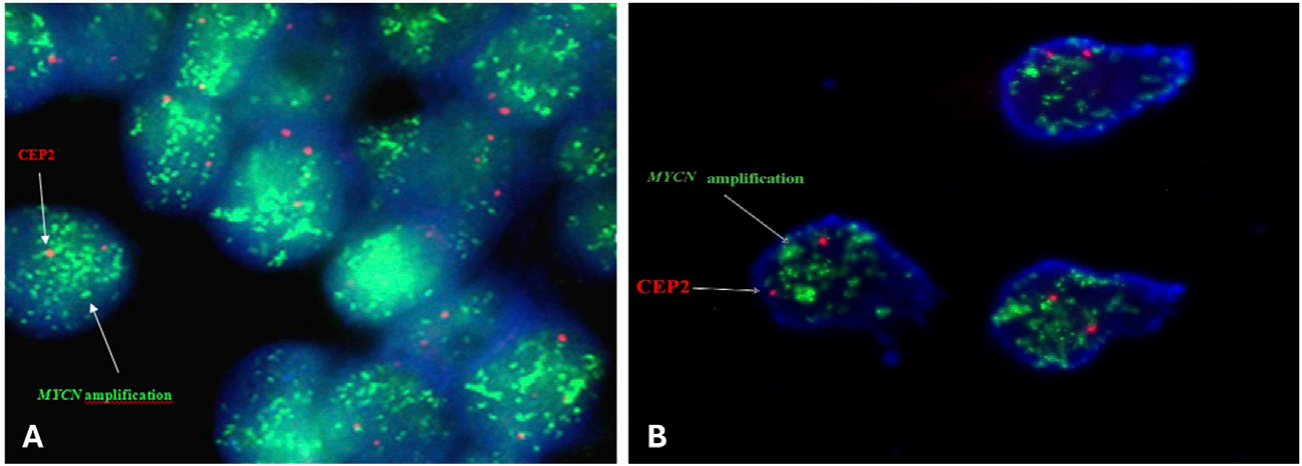
Representative FISH image on paraffin-embedded tissue section **(A)** and intraoperative tumor imprints **(B)** using Vysis LSI N-MYC (2p24) SpectrumGreen/CEP 2 SpectrumOrange Probe from a neuroblastoma tumor showing amplification MYCN captured with Applied Spectral Imaging- ASI- Israel at ×100 magnification on the Nikon epifluorescence microscope.

Positive results for *MYCN* amplification were observed in twelve patients’ paraffin-embedded tissue sections and in three patients’ intraoperative tumor imprints which represents 22.4% of all tested patients in the analysis (Figure 2).

There were 9 (60%) little boys and 6 (40%) little girls included in the study. Of the 15 patients, 1 (6.7%) had stage 3 disease, 1 (6.7%) had stage 4 s disease, and 13 (86.6%) had stage 4 disease. Of these 15 patients with MYCN amplifications 5 (33.3%) were <18 months of age, and 10 (66.7%) were >18 months of age. Five (33.3%) of the patients with MYCN amplification had a tumor mass in the abdomen and ten (66.7%) had a tumor mass in the adrenal gland.

### Molecular karyotype results

To evaluate additional molecular abnormalities we performed microarray testing on 6 patients with different stages of disease, tumor localization and ages at diagnosis.

**FIGURE 3:**
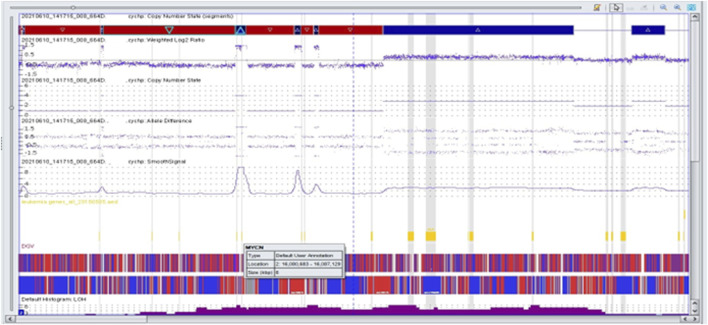
Complex copy number gains and losses in the region 2p25.3p21 of chromosome 2 in patient 63. With Neuroblastoma by single-nucleotide polymorphism (SNP) array. Colored segmental regions (top line: red color—deletions; blue color—gains), copy number status, allelic difference, and smooth signal aberrations by SNP array.

**FIGURE 4:**
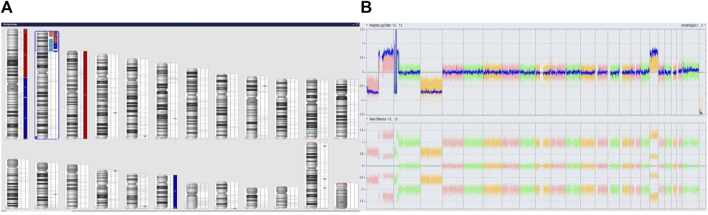
Complex copy number gains and losses in the whole genome of patient 63. With Neuroblastoma. Colored segmental regions (top line: red color—deletions; blue color—gains) **(A)** and Whole Genome View **(B)**.

We performed microarray testing on a patient with stage 4 disease and tumor localization in the adrenal gland. The patient was a girl who was over 18 months of age at the time of diagnosis. Through fluorescence *in situ* hybridization we observed positive results for *MYCN* amplification. Microarray analysis revealed a deletion within the short arm of chromosome 1 (arr [GRCh37] 1p36.33p36.22 (849466_9304205)x1), and a duplication involving a fragment of the short arm of chromosome 1, and the long arm of chromosome 1 (arr [GRCh37] 1p13.3q44 (110062169_249224684)x3). In region 2p25.3p21 we observed chromothripsis (arr [GRCh37] 2p25.3p21 (12770_46642249)cth) (Figure 3). The *MYCN* gene (2p24.3) was amplified in this region (10 copies). In addition, the *ALK* gene (2p23.2-p23.1) was also duplicated. Monosomy of chromosome 3 and trisomy of chromosome 18 were identified (Figure 4).

**FIGURE 5:**
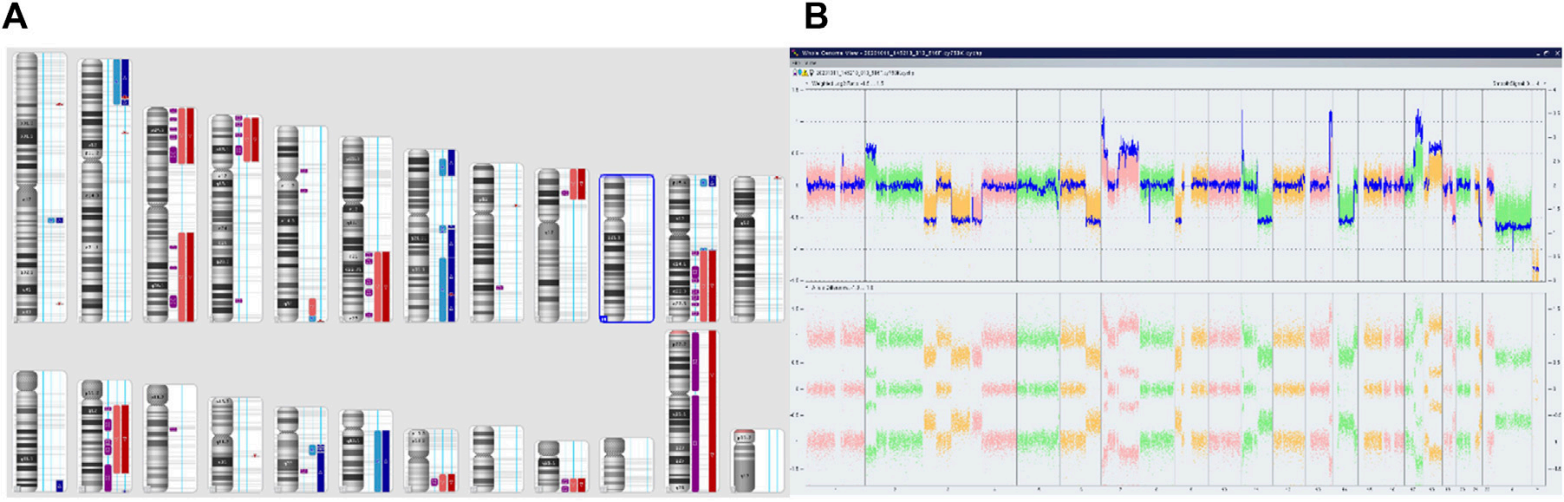
Complex copy number gains and losses in the whole genome of patient 64 with Neuroblastoma. Colored segmental regions (top line: red color—deletions; blue color—gains) **(A)** and Whole Genome View **(B)**.

The second patient was a boy over 18 months of age at diagnosis, with stage 4 disease and tumor localization in the adrenal gland. Through fluorescence *in situ* hybridization we did not observe any amplification of the *MYCN* gene. Microarray analysis identified 3 cell lines with various genetic changes (Figure 5). We present the results in the table below (Table 2).

**TABLE 2:** Molecular changes identified in the microarray analysis of patient 64.

	Molecular karyotype	
	Different aberrations	Common aberrations
First cell line	arr [GRCh37] 1q21.3q23.1 (153001296_157812399)x3 [0.7], 2p25.3p21 (12771_42229545)x3 [0.7], 3p26.3p21.2 (61892_51747702)x1 [0.7], 3q13.31q29 (115403680_197851444)x1 [0.7], 4p16.3p13 (2744163_43086625)x1 [0.7], 6q21q27 (106487938_170914297)x1 [0.7], 7p21.3p15.3 (8582953_24331799)x3 [0.7], 7q11.22q36.3 (70144228_159119707)x3 [0.7], 9p24.3p21.1 (208455_28198623)x1 [0.7], 11p15.4 (3189610_10187920)x3 [0.7] 11q13.3q25 (69472473_134937416)x1 [0.7] 14q11.2q31.3 (24238818_89633472)x1 [0.7], 17q12q21.32 (36152227_46070727)x3 [0.7], 18q11.2q23 (19414307_78013728)x3 [0.7], 19q13.2q13.43 (42791601_58956816)x1 [0.7], 21q22.11q22.3 (35184499_48093361)x1 [0.7]	arr [GRCh37] (Y)x0, 7p22.3p21.3 (43377_8573211)x3∼4, 11p15.5p15.4 (230681_3021529)x3∼4, 13q33.1q34 (103826264_115107733)x3∼4 14q31.1q32.33 (81449690_107279475)x2hmz 17q21.32q25.3 (46073099_81041823)x3∼4
Second cell line	arr [GRCh37]11q13.2q13.3 (66814096_69464793)x3 [0.4]	
Third cell line	arr [GRCh37]5q33.3q35.2 (159230740_174855411)x1 [0.25], 5q35.2q35.3 (174856439_180715096)x3 [0.25]	

The third patient was a girl who was over 18 months of age at diagnosis, with stage 3 disease and tumor localization in the abdomen. Through fluorescence *in situ* hybridization we did not observe any amplification of the *MYCN* gene. Microarray analysis revealed mosaic monosomy of chromosome X and duplication of region 22q11.21 (arr [GRCh37] 22q11.21 (18979347_21800471)x3). The Whole Genome View (WGV) showed a decrease in the log2ratio value, which may indicate the presence of monosomy on chromosomes 3, 4, 9, 10, 14, 15, 19, and 21, and an increase in the log2ratio value, which may indicate the presence of trisomy on chromosome 13. An ambiguous assessment of chromosome copy number may be the result of incorrect material collection. The fourth patient was a boy who was over 18 months of age at diagnosis, with stage 3 disease and tumor localization in the abdomen. *MYCN* amplification was negative. With microarray analysis we identified a duplication involving a fragment of the short arm of chromosome 1 (arr [GRCh37]1p36.33p36.32 (849467_3627839)x3 [0.3]) and deletions in the following regions: arr [GRCh37]12q24.23q24.31 (118233114_125556278)x1 [0.2], 19p13.3p11 (260912_24505049)x1 [0.2]. As with the previous patient the Whole Genome View (WGV) showed a decrease in the log2ratio value, which may indicate the presence of monosomy on chromosomes 3, 4, 9, 10, 14, and 15 and an increase in the log2ratio value, which may indicate the presence of trisomy on chromosome 13. The microarray results for the two patients were normal and did not reveal any additional changes in the molecular karyotype.

## Discussion

In recent years, significant progress has been made in understanding the genetic basis of neuroblastoma. Numerous studies have been published on somatic mutations and chromosomal abnormalities associated with the development of neuroblastoma. However, their role in treatment response, prognosis, and the development of metastasis has not been fully elucidated. Based on our research, we have divided patients into two groups: those with *MYCN* amplification and those without it.

High-risk patients are identified by the presence of *MYCN* amplification. *MYCN* amplification is a powerful prognostic factor for advanced stages and rapid tumor progression (Lau et al., 2015). Interphase FISH analysis provides a direct and rapid method. Only a small number of tumor cells are required, compared with primary tumor biopsies, and FISH is more sensitive by counting the *MYCN* copy number in each single cell (Tibiletti, 2007; Campbell et al., 2017).

The fluorescence *in situ* hybridization (FISH) method is faster to prepare than molecular biology techniques or traditional cytogenetic analysis. FISH can be used on interphase nuclei, in contrast to karyotype analysis, and it can be performed on formalin-fixed nuclei, in cases where a fresh or frozen tumor is not available (e.g., small biopsies or retrospective studies). However, molecular methods, such as microarrays, have strong limitations when analyzing *MYCN* amplification and other genetic changes in neuroblastoma because molecular methods are based on DNA from a mixture of cells and therefore only provide an average result for a particular tumor. When a tumor is highly amplified, this is not a problem for diagnosis. However, with low levels of *MYCN* amplification, it can be difficult to interpret the results. The first possibility is that there is a heterogeneous population of tumor cells in which a small number of cells are highly amplified or have other anomalies. Or it may be a highly amplified tumor mixed with normal tissue. Finally, it may be a low level of amplification occurring in tumor cells (e.g., a triploid population) (Shapiro et al., 1993; Squire et al., 1996; Thorner et al., 2006).

In the Polish population, tumor imprints are routinely used to assess *MYCN* amplification. However, this is not always possible and then the only material for *MYCN* amplification is a paraffin tissue section. It can be done on both fresh and archived tissues from many years ago.

For this study we used intraoperative tumor imprints and fresh paraffin-embedded tissue sections in addition to archival materials. Fresh material was obtained from a tumor removed from a patient 2 weeks before the study and archival paraffin sections were removed from a patient operated on 8 years and 2 months before the study. In our opinion both fresh tissue and tissue from years before preserved in a paraffin block were adequate materials for the test. The quality of the specimens was similar and the evaluation of both archival and fresh specimens was consistent with previous studies. Intraoperative tumor imprints were taken immediately after their removal. The quality of these specimens is much better compared to the paraffin-embedded tissue sections, as only the tumor cells are on the slide, with no surrounding tissue. The assessment of *MYCN* amplification on intraoperative tumor imprints does not cause any diagnostic problems.

Microarray testing of neuroblastoma samples provides additional information on other chromosomal changes observed in patients with and without *MYCN* amplification, which has a strong impact on prognosis. In a sample from a patient with *MYCN* amplification we found a deletion within the short arm of chromosome 1 (1p36.33p36.22), and a duplication involving a fragment of the short arm of chromosome 1 and the long arm of chromosome 1 (1p13.3q44). In the literature, deletion or loss of heterozygosity (LOH) of the 1p36 chromosomal band is correlated with high-risk neuroblastoma features, including older age at diagnosis, metastatic disease, *MYCN* amplification and poor prognosis Unfavorable abnormalities also include segmental copy number alterations, such as loss of 3p, deletion or LOH 11q23, 14q23qter and gain of 17q (Marrano et al., 2017). Amplification of the *MYCN* gene was observed only at a level of 10 copies. Unfortunately, this can be considered a limitation of the molecular method because the FISH analysis showed the amplification of the *MYCN* gene at a level of approximately 30 copies. In our opinion, the gold standard for the assessment of *MYCN* amplification is fluorescence *in situ* hybridization on intraoperative tumor imprints. Furthermore, microarray analysis showed a duplication of the 2p23.2p23.1 region, including the *ALK* gene. Anaplastic lymphoma kinase (ALK) is an important regulator of stem cell functions, including STAT3 dependent self-renewal, and as a transcriptional target of *MYCN*, high expression indicates an unfavorable prognosis (Rosswog et al., 2023). According to studies carried out on genetically engineered mouse models of NB, the *MYCN* and *ALK* genes cooperate to promote oncogenesis (Vivancos Stalin et al., 2019). Additionally, CytoScan HD analysis revealed a monosomy of chromosome 3 and a trisomy of chromosome 18.

In 3 out of 5 patients without changes in *MYCN* amplifications, we observed complex alterations in the microarray pattern. In two samples, discrete changes indicating the presence of monosomy of chromosomes 3, 4, 9, 10, 14, and 15. According to scientific research chromosome 10 is the most frequent loss. On the long arm of chromosome 10, there are tumor suppressor genes such as *PTEN*, *DBMT*, and *LGI1*, while the short arm contains *IDI1*, *AKR1C3*, *DDH1*, *NET1A*, *PRKCQ*, and *GATA*-binding protein 3. Among these, *PTEN* is the second most commonly deleted/mutated gene in human tumors, with a mutation rate of 50%. The GDNF family receptor alpha 2 (GFRA2) promotes the proliferation of NB cells (Li et al., 2019). According to the literature, patients with neuroblastoma and an abnormal chromosome 10 had a significantly lower 3-year OS compared to the group of patients with a normal copy number of chromosome 10 (Jiang et al., 2021). In the same samples we noted an extra copy of chromosome 13, which, according to other researchers (Bilke et al., 2008; Ambros et al., 2014) is a common chromosome in the trisomy of neuroblastoma. Interestingly, some authors show that there are no structural changes on chromosome 13 in neuroblastoma (Bogen et al., 2016). While its prognostic significance in neuroblastoma has not been precisely determined, in other cancers, including ALL, AML, and MDS, trisomy 13 is closely associated with an unfavorable prognosis for the patient. The presence of trisomy 13 in acute leukemia cells indicates a poor response to chemotherapy (Spirito et al., 2003; Herold et al., 2014). Monosomy of chromosome X and duplication of region 22q11.21 have not been described in the literature in patients with neuroblastoma so far. However, our results unequivocally demonstrate that such alterations may also be present in this type of cancer.

According to the Polish “Standards for the genetic diagnostics of solid tumors in children” structural changes involving chromosomal regions 1p, 1q, 2p, 3p, 4p, 6p, 6q, 11q, and 17q are most commonly observed in advanced stages of the disease in older children and represent unfavorable prognostic factors, increasing the risk of disease recurrence, regardless of the specific chromosomal pairs affected (Trubicka et al., 2023). In one of our patients with stage 4 disease, the FISH study found no amplification of the MYCN gene, while the microarray analysis identified 3 cell lines in which some of the above-mentioned structural changes were noted (including: 1p, 2p, 3p, 4p, 6q, 11q, and 17q) stratifying the patient into a group with an unfavorable prognosis.

In the literature, high-risk patients had long-term survival rates of only 10%–20% with combination chemotherapy, surgery, and local radiation therapy (Tolbert and Matthay, 2018). However, treatment approaches that used a combination of induction therapy, myeloablative consolidation therapy with stem cell support, and biological therapy had improved 5-year survival rates from less than 15%–40% (Nakagawara et al., 2018).

Despite many biochemical, molecular and genetic indicators as markers of poor prognosis, *MYCN* amplification is still used to stratify patients into risk groups and is a marker of poor prognosis. These patients respond poorly to conventional therapy. The ability to determine this biological marker is necessary for appropriate clinical management. Unfortunately, treatment failure has been reported in all patient groups, even without *MYCN* amplification, suggesting that additional prognostic markers must be discovered to improve treatment protocols (Feng et al., 2023).

In conclusion, both the FISH technique and microarray testing have their advantages and disadvantages. In addition to *MYCN* gene amplification, cytogenetic abnormalities are also a marker of an unfavorable prognosis in patients diagnosed with neuroblastoma. Therefore, it is very important that the result be based on both, fluorescence *in situ* hybridization and molecular karyotype.

## Data Availability

The original contributions presented in the study are included in the article/Supplementary Material, further inquiries can be directed to the corresponding authors.
