# Supplementary material for: Application of the FISH method and high-density SNP arrays to assess genetic changes in neuroblastoma—research by one institute
Source: Acta Biochim Pol. 2024 Jul 10;71:12821. doi: 10.3389/abp.2024.12821 (PMC11267511; doi:10.3389/abp.2024.12821)
Supplement: Supplementary file 1 [file Table1.DOCX]

Table. Detailed patient data.

| **Patient** | **Age at diagnosis** | **Diagnosis** | **INSS risk group** | **Primary site** | ***MYCN* amplification** |
| --- | --- | --- | --- | --- | --- |
| 1. | <18 m | NB | 3 | pelvis | No |
| 2. | >18 m | NB | 4 | abdomen | No |
| 3. | <18 m | GNB | 4 | abdomen | No |
| 4. | >18 m | GNB | 4 | abdomen | No |
| 5. | >18 m | NB | 4 | abdomen | No |
| 6. | >18 m | NB | 4 | mediastinum | No |
| 7. | >18 m | GNB | 3 | adrenal | Yes |
| 8. | <18 m | GNB | 1 | pelvis | No |
| 9. | <18 m | NB | 4 | adrenal | Yes |
| 10. | <18 m | NB | 3 | pelvis | No |
| 11. | >18 m | NB | 4 | abdomen | Yes |
| 12. | <18 m | GNB | 3 | mediastinum | No |
| 13. | <18 m | NB | 2 | mediastinum | No |
| 14. | >18 m | GN | 3 | mediastinum | No |
| 15. | <18 m | NB | 4 | pelvis | No |
| 16. | >18 m | NB | 2 | mediastinum | No |
| 17. | >18 m | NB | 3 | abdomen | No |
| 18. | >18 m | NB | 4 | abdomen | No |
| 19. | <18 m | NB | 3 | adrenal | No |
| 20. | >18 m | NB | 4 | abdomen | No |
| 21. | <18 m | NB | 3 | mediastinum | No |
| 22. | <18 m | NB | 1 | adrenal | No |
| 23. | >18 m | NB | 4 | abdomen | No |
| 24. | <18 m | NB | 3 | mediastinum | No |
| 25. | <18 m | NB | 4s | cervical | No |
| 26. | <18 m | NB | 4 | adrenal | Yes |
| 27. | >18 m | GNB | 3 | mediastinum | No |
| 28. | <18 m | ND | 3 | mediastinum | No |
| 29. | >18 m | NB | 4 | abdomen | No |
| 30. | >18 m | NB | 4 | adrenal | Yes |
| 31. | >18 m | NB | 3 | pelvis | No |
| 32. | >18 m | NB | 1 | mediastinum | No |
| 33. | >18 m | NB | 4 | adrenal | No |
| 34. | >18 m | NB | 3 | adrenal | Yes |
| 35. | >18 m | NB | 3 | abdomen | No |
| 36. | >18 m | ND | 4 | adrenal | Yes |
| 37. | <18 m | NB | 4s | adrenal | Yes |
| 38. | >18 m | NB | 4 | adrenal | Yes |
| 39. | <18 m | NB | 3 | adrenal | No |
| 40. | <18 m | ND | 1 | mediastinum | No |
| 41. | <18 m | NB | 1 | adrenal | No |
| 42. | <18 m | NB | 3 | pelvis | No |
| 43. | <18 m | NB | 4 | adrenal | No |
| 44. | >18 m | NB | 4 | adrenal | Yes |
| 45. | >18 m | GNB | 1 | adrenal | No |
| 46. | >18 m | NB | 3 | abdomen | No |
| 47. | <18 m | NB | 3 | pelvis | No |
| 48. | >18 m | GNB | 4 | adrenal | No |
| 49. | <18 m | NB | 4s | adrenal | No |
| 50. | <18 m | NB | 3 | adrenal | No |
| 51. | >18 m | GNB | 4 | adrenal | No |
| 52. | >18 m | NB | 3 | abdomen | No |
| 53. | >18 m | NB | 4 | abdomen | Yes |
| 54. | <18 m | NB | 4 | adrenal | No |
| 55. | <18 m | NB | 4 | abdomen | Yes |
| 56. | <18 m | NB | 4 | adrenal | No |
| 57. | >18 m | GNB | 4 | abdomen | No |
| 58. | >18 m | NB | 4 | abdomen | Yes |
| 59. | >18 m | NB | 3 | abdomen | No |
| 60. | <18 m | NB | 1 | adrenal | No |
| 61. | <18 m | NB | 3 | cervical | No |
| 62. | <18 m | NB | 4 | abdomen | Yes |
| 63. | >18 m | NB | 4 | adrenal | Yes |
| 64. | >18 m | NB | 4 | adrenal | No |
| 65. | >18 m | NB | 3 | abdomen | No |
| 66. | <18 m | NB | 2 | abdomen | No |
| 67. | >18 m | GNB | 3 | abdomen | No |
